# General approaches to ethical reasoning in Islamic biomedical ethics discourse

**Published:** 2018-09-09

**Authors:** Hamideh Moosapour, Jannat Mashayekhi, Farzaneh Zahedi, Akbar Soltani, Bagher Larijani

**Affiliations:** 1 *Ph.D Candidate in Philosophy of Medicine and Medical Ethics, Evidence Based Medicine Research Center, Endocrinology and Metabolism Clinical Sciences Institute, Tehran University of Medical Sciences, Tehran, Iran* *.*; 2 *Ph.D Candidate in Medical Ethics, Medical Ethics and History of Medicine Research Center, Tehran University of Medical Sciences, Tehran, Iran; Department of Medical Ethics, School of Medicine, Tehran University of Medical Sciences, Tehran, Iran.*; 3 *Director of* *Medical Ethics Research Group, Endocrinology and Metabolism Research Center, Endocrinology and Metabolism Clinical Sciences Institute, Tehran University of Medical Sciences, Tehran, Iran.*; 4 *Professor, Evidence Based Medicine Research Center, Endocrinology and Metabolism Clinical Sciences Institute, Tehran University of Medical Sciences, Tehran, Iran* *.*; 5 *Professor, Endocrinology and Metabolism Research Center, Endocrinology and Metabolism Clinical Sciences Institute, Tehran University of Medical Sciences, Tehran, Iran* *.*

**Keywords:** *Ethical reasoning*, *Islamic biomedical ethics*, *Islamic biomedical ethics principles*, *Ethical decision-making*

## Abstract

Islamic and non-religious ethics discourses have similarities and differences at the levels of meta-, normative, and applied ethics (e.g. biomedical ethics). Mainstream biomedical ethics (MBME) uses the language of contemporary, non-religious, Western ethics. Significant effort has been dedicated to comparing Islamic biomedical ethics (IBME) and MBME in terms of meta- and normative ethical positions, and final decisions on practical ethical issues have been reached. However, less attention has been given to comparing the general approaches of the two aforementioned discourses to ethical reasoning. Furthermore, IBME uses different languages to approach ethical reasoning, but identification and conceptualization of these approaches are among the important gaps in the literature. The aim of this study was to conceptualize general approaches to ethical reasoning in IBME. Through review and content analysis of the existing literature and the comparison between the languages employed by IBME and MBME, an inductive distinction have been made. The languages used in conceptualized approaches include the followings: (*i*) a language in common with the one employed by MBME; (*ii*) MBME language adjusted to the basic, common beliefs of Muslims; (*iii*) a language based on *fatwas*; and (*iv*) a language based on IBME principles. In the authors’ opinion, major challenges of the above-mentioned four approaches include, respectively: identifying the lack of religious sensitivity or Islamic considerations regarding an issue; acknowledging specific beliefs as the basic, common beliefs of Muslims; diverse *fatwas* and relations between juridical soundness and ethical soundness; and agreement on the same principles and rules as well as who should apply them.

## Introduction

Ethical reasoning is a practical reasoning about what moral agents should do. Ethical arguments, like all other arguments, have the structure or form apart from the content (i.e., a conclusion and its supporting premises). General structures of ethical reasoning in biomedicine have been mainly adopted from the discipline of philosophy. The three main levels of the philosophical study of ethics include meta-ethics, normative ethics, and applied ethics. In biomedical ethics as a branch of applied ethics, reasoning depends mainly on the meta- and normative ethical assumptions of the decision maker. This dependency also exists in the case of reasoning in Islamic biomedical ethics (IBME) (1). 

Similarities and differences between Islamic and non-religious ethics in meta- and normative ethical assumptions lead to the similarities and differences between the two discourses in ethical reasoning about biomedical issues (2).The discipline of biomedical ethics has taken its form and content from contemporary, non-religious, Western ethics to shape the mainstream biomedical ethics (MBME). An extensive body of literature on IBME compares fairly diverse Islamic positions and their supporting arguments with non-religious ones. Moreover, a vast amount of literature exists on laws and regulations in Muslim-majority countries, and exists on slightly different juridical positions on sensitive issues. In addition, subjects such as Islamic meta-ethical and normative ethical positions, methods employed in Islamic jurisprudence (*Fiqh*), and basic Islamic concepts similar to the main concepts of MBME are extensively discussed in the literature. However, an important gap exists in the literature on different approaches to ethical reasoning in IBME, i.e., regarding how ethicists reason (or should reason) about biomedical issues in Islamic discourse (2). Moreover, it seems that Islamic biomedical ethicists use different languages for ethical reasoning, for example a language similar to non-religious biomedical ethics, or to that of Islamic Jurisprudence. The aim of the this study was to identify and conceptualize general approaches to the ethical reasoning utilized in IBME discourse.

## Method

In this study, we reviewed and analyzed relevant literature on IBME, and then made an inductive distinction of the languages used for ethical reasoning in this discourse. This review was not systematic by definition; however, the authors searched PubMed and Google Scholar databases for combinational keywords from the following categories: 1) Islamic, Islam, Shi’a, Shiite, Shi'ite  or Sunni, 2) biomedical ethics, medical ethics, or bioethics, and 3) method, approach, reasoning, thinking, process, debate, epistemology, or philosophy. The distinction was conceptualized by comparison between the languages of IBME and MBME. Additionally, the authors provided several main challenges confronting each approach, though a comprehensive discussion of all the challenges was beyond the scope of this work. The results of our work have been organized in the present article as follows:

Firstly, a short overview of the most important approaches to ethical reasoning in MBME is provided. These approaches have often been the issue of comparison between IBME and MBME in relevant literature. Secondly, four approaches of IBME conceptualized in this work are briefly presented. Thirdly, each approach is detailed with examples, and subsequently the challenges of each approach are discussed. Lastly, a conclusion is provided.

**Approaches to ethical reasoning in MBME**

The discipline of biomedical ethics has a rich history of approaches to ethical reasoning. While the principlist and utilitarian approaches of English language biomedical ethics are the leading parts of the mainstream, there is considerable interest in different alternatives contributing to enrichment of the area. Instances include casuistry, virtue ethics )which is enjoying a strong upsurge and renewal of interest(, deontology, empirical ethics as an impressive turn in the field, hermeneutic perspectives, reflective equilibrium, narrative ethics, feminist ethics, pragmatist approaches, communitarianism, deliberative ethics, European philosophy approaches such as phenomenological perspective, existentialism, and ethics of care (3, 4). 

Philosophy provides valuable approaches to ethical reasoning in biomedical ethics. These approaches have been analyzed, classified, and evaluated from different perspectives. According to the position that may be taken on meta- and normative ethical problems, a distinction could be made among the most important philosophical approaches to ethical reasoning by answering these questions:

     *(i)   *Where should we seek right-making features (or characteristics that make an action right) to make an ethical decision?

    *(ii)*   Which features of the action make it ethically right?

     *(iii)   *How many right-making features are necessary to form sound bases for ethical reasoning?

Answers to the first question have developed three well-known types of ethical theories: virtue, deontological, and consequentialist ethics. Virtue theories guide and assess the character traits of a moral agent; in contrast, theories of deontology and consequentialism guide and assess the action we ought to take depending on the action adherence to ethical obligations or consequences of that particular action, respectively (1).

The next two questions are pertinent to the nature and number of right-making features in ethical reasoning. Their answers develop normative ethical theories that seek not only to determine the right acts, but also to identify their properties. To take well-known examples, the right-making feature in utilitarian theories is utility. These theories have an absolute supreme principle saying that the right action is one that maximizes the balance of good consequences over bad ones (1). The ‘four principles approach’, on the other hand, is an example of a theory with multiple principles or right-making features. It is developed by Beauchamp and Childress. In this theory, each of the four principles (respect for patients’ autonomy, beneficence, non-maleficence, and justice) introduces a right-making feature (5). Ross's ethical theory as another example lists seven right-making features for ethical reasoning as prima facie duties including fidelity, reparation, gratitude, beneficence, non-maleficence, self-improvement, and justice (6). 

**General approaches to ethical reasoning in IBME**

In this section, four approaches of IBME conceptualized in this work are briefly presented at first, and then each approach is detailed with examples. The approaches are as follows: 

     1)   Ethical reasoning using a language in common with MBME language 

     2)   Ethical reasoning using MBME language adjusted to the basic, common beliefs of Muslims 

     3)   Ethical reasoning using a language based on *fatwas*

     4)   Ethical reasoning using a language based on IBME principles

For most biomedical issues, non-religious sensitivity is considered; hence, a language in common with MBME language is generally used in ethical reasoning for those issues in IBME (approach 1). For example, ethical reasoning about professional commitments such as honesty or altruism is independent from religious beliefs (7). However, this approach is not sufficient for religiously sensitive issues such as advance directive, organ transplantation or abortion. 

In the case of some religiously sensitive issues, there is general recognition that it is not necessary to seek relevant *fatwa(s)*, i.e., juridical opinions issued by chosen qualified scholars in Islamic jurisprudence (*Marja’ Taqlid*^1^ or* Mufti*^2^) for ethical reasoning. In such cases, Islamic considerations in ethical reasoning are based on the basic, common beliefs of Muslims, but not necessarily on *fatwas* (approach 2). For instance, arguments concerning the ethical aspects of advance directive in IBME may be based on a language in common with MBME language, e.g., the four principles approach (5). Nevertheless, the principle of 'respect for patients’ autonomy' cannot be applied in IBME as in MBME; this principle is adjusted to a customized version because of the limited definition and scope of autonomy in Islamic beliefs (8). 

By contrast, Muslims (including Islamic ethicists) believe that it is essential to seek and follow a *fatwa* to gain an Islamic perspective on religiously sensitive issues such as organ transplantation or abortion; consequently, ethical reasoning in this case will be in line with a given *fatwa* to put forward a convincing argument. For example, *fatwas *of the supreme leader on therapeutic abortion (TA) and assisted reproductive technologies (ARTs) have made those practices legitimate in Iran (9 - 11).

Some authors have proposed using a principle-rule based language in IBME (12 - 16). The principles and rules of the ethico-legal reasoning employed by Islamic jurists have the potential for applying to ethical reasoning in IBME. For example, the rule of necessity allows a Muslim to choose a lesser harm over a greater one, and therefore this rule allows the mother to prefer her own safety to that of the fetus in a maternal life-threatening situation. Hence, abortion is not unethical from an Islamic point of view in this situation (12).

Moreover, we emphasize that this classification is based on the conceptual distinctions made for a theoretical, heuristic purpose. Hence, developing arguments or making decisions on a specific subject in practice or in IBME literature is not restricted to just one approach. However, a combination of approaches is usually applicable to most issues. 

This is like other formal distinctions where conceptually distinct things always exist alongside one another in reality.

^1^ In *Shiite* schools of Islamic jurisprudence

^2^ In *Sunni* schools of Islamic jurisprudence

*Approach 1: Ethical Reasoning Using a Language in Common with MBME Language*

A language in common with MBME language is often used for ethical reasoning in IBME. Examples are found in issues such as medical professionalism, healthcare resource allocation, research on animals, and research on human subjects. Decisions or ethical considerations about these issues in IBME (as reflected in ethical codes, for instance) are generally the same as those of MBME (7, 17 - 19). However, the philosophy behind IBME positions in these cases may be different from those of MBME. In addition, the religious orders or recommendations on these issues not only provide guidance on relevant ethical considerations, but also specify divine rewards and punishments guaranteeing that those ethical codes are obeyed faithfully and without external supervision (20, 21). 

*Approach 2: Ethical Reasoning Using MBME Language adjusted to the Basic, Common Beliefs of Muslims*

For some morally and religiously sensitive issues such as euthanasia, living will, advance directive, informed consent in children and other similar issues, ethical arguments unavoidably involve the basic, common beliefs of Muslims such as eternity of life (the immortal soul and life after death), and seeking perfection. Hence, ethical reasoning in these cases may employ a language in common with MBME language, but it is necessary to adjust this language to the basic, common beliefs of Muslims (22 - 25).

Some authors have reflected on IBME to seek the main concepts of MBME in Islamic resources, and explore the differences between IBME and MBME from this perspective. Good examples can be found in studies explaining ethical reasoning in Islam in terms of important concepts in western moral philosophy, e.g., the three theories of virtue, deontological, and consequentialist ethics, or studies making interpretations of MBME theories, for example the four principles approach, based on Islamic beliefs. 

As regards these three well-known ethical theories, IBME seems to be mainly based on duties and obligations at first glance, whereas MBME seems more based on rights, particularly those of the individual (26, 27); however, IBME uses all three approaches mentioned above, i.e., virtue, deontological, and consequentialist ethics. IBME literature contains many arguments based on the concepts of principles, duties, rights, consequences, or virtues (15, 28). 

Regarding deontological ethics, Muslims evidently try to find moral obligations in *Sharia *sources because of a religious, moral duty to obey God’s orders*. *The moral obligation to save patients’ lives to preserve human dignity is such an example.

Regarding consequentialist ethics, the important principle of the common good or public interest (*Maslaha)* evidently has a consequentialist view. When the authoritative hierarchy of Islamic resources does not provide God’s command to His followers about a definite issue, *Maslaha* can be used to approach that issue, and consequently incorporate new issues into the Muslim community (12, 13). Ethical reasoning about therapeutic cloning and embryonic stem cell research are examples for which consequentialist arguments such as weighing the benefits to society were made by jurists (28). 

Regarding virtue ethics, professional medical ethics in the Islamic tradition known as *Adab *or *Akhlaq* represents the tradition of virtue ethics in Islam. For instance, *Firdaus al-Hikma fi al-Tibb *by Ali bin Sahl Rabban at-Tabari or *Adab al-Tabib *by Al-Ruhawi emphasize virtue ethics in physicians’ practice, which could resolve or prevent numerous moral dilemmas in medicine. As Weber said, the Islamic tradition has emphasized moral virtues in the practice of medicine to the extent that the criteria for a virtuous physician in Islam are analogous to those for a reliable legal witness in *Sharia* law. These criteria include being a) an adult Muslim, b) sane, c) unbiased, d) and *‘adil*, i.e., having a good and blameless reputation (28). Due to the importance of reliable legal witnesses in *Sharia* law, Islamic ethics demands that physicians have high standards of moral virtues. 

In recent years, the four principles approach has been extremely prominent while general approaches to ethical reasoning in MBME have been the subject of much debate. The principles are ‘respect for patients’ autonomy‘, ‘beneficence’, ‘non-maleficence’ and ‘justice’. This set of moral principles provides a starting point for resolving ethical problems in biomedicine (5). Hence, a number of studies have tried to seek the main concepts of the four principles approach in Islamic resources and explore the differences between IBME and MBME from this perspective (29). These studies demonstrate that the roots of the four principles are evident in Islamic resources; however, there are some differences in the application of these principles in ethical reasoning, especially in the case of autonomy. 

On the one hand, significant room exists for autonomy in Islam. Considering human beings as God’s vicegerents on earth, the importance of ‘*ilm’ *(knowledge) in making reasoned decisions, humans’ direct accountability for their actions before God without the mediation of a clergy, and their free will to either accept or reject the divine command are proofs for the importance of individual autonomy in Islam. According to Islamic teachings, one must surrender only to God and to no other creature and that is the definition of *Abd *(29). On the other hand, a feeling of responsibility toward God limits the personal choices of a devout Muslim as some actions are forbidden by *Sharia*. Another limitation of autonomy for Muslim patients is the importance of social coherence where the family, relatives and public interest influence the patients’ decisions (26, 30). In Muslim societies, the principle of public interest (*Maslaha*) and the principle of justice take priority over autonomy as the collective interest takes priority over the benefits of individuals. It seems that this value may be rooted in the spirit of Islam or in the culture of Islamic societies mainly in Asia, Africa, and the Middle-East or both of them (31).

*Approach 3: Ethical Reasoning Using a Language Based on Fatwas*

In Islamic discourse, rightness or wrongness of an ‘action’ is traditionally discussed in the field of ‘Islamic jurisprudence’ (*Fiqh*) rather than in ‘Islamic ethics’. The former is more established, rigorous and disciplined than the latter. The field of ‘Islamic ethics’ is mostly focused on the ‘moral agent’ to promote his or her ethical virtues. Spiritual growth and striving to be a perfect human depend not only on obeying the juridical obligation, but also on promoting ethical virtues. In comparison with Islamic discourse, rightness or wrongness of an ‘action’ in modern, non-religious discourse is generally discussed in the field of ‘ethics’; likewise, rightness or wrongness of biomedical practice is discussed in the field of biomedical ethics. 

When Muslim patients or Muslim healthcare providers face a medical issue not fully compatible with the general guidance of *Sharia *(especially about medical issues of higher ethical and religious sensitivity), they seek the relevant *fatwas* to obtain the Islamic perspective on that issue. For instance, when a continued pregnancy endangers the mother's life and therapeutic abortion is an available option, the Muslim mother or her doctor seeks relevant *fatwas* on the case. They need to know whether abortion is ethically and religiously justified under the circumstances, or whether preferring the mother’s life to the fetus’ life is ethically and religiously right. Ethical reasoning regarding such issues is mainly based on relevant *fatwas*. 

A* fatwa* is a Muslim jurist’s perspective on a definite subject inferred from Islamic resources and issued in the form of a legal opinion to respond to and guide his followers. However, medical *fatwas* are not so numerous that they can cover all questions in IBME, and they may be applied when: 1) it is crucial to seek the Islamic judgment on the issue at hand due to its extreme religious and moral sensitivity for Muslims, and 2) reaching the Islamic judgment is not easy and straightforward due to the complexity of the situation (unlike approach 2). 

*Fatwas* have two main functions at individual and social levels. At the individual level *fatwas* define the follower’s (*Mukalaf’s)* religious duty, which he or she has an inner obligation to fulfill. At the social level, they have great potential to make a concern legitimate in Muslim societies. *Fatwas* are not legally obligatory, although in Muslim-majority countries current *fatwas* have affected the laws directly and indirectly. *Fatwas* play two roles in the laws of these countries. First, they are followed by physicians and patients when the national laws are silent about a medical procedure or technology. Second, they are used to provide an opportunity to make certain sensitive practices socially and then legally legitimate (9, 10, 32-35). Legitimacy of assisted reproductive technologies (ARTs) in Iran could be a good example. Ayatollah Khamenei, the supreme leader of Iran and a qualified religious scholar, issued a* fatwa* in 1999 in response to increasing public demand for infertility treatments. His statement permitted some ART methods including the use of donated embryos or donated gametes of third parties in the process of assisted reproduction for infertile families. After ARTs were justified non-officially through the *fatwa *of the supreme leader, the ensuing practice initiated a more official pathway in the case of embryo donation to approve a relevant act by the parliament: the Iran Embryo Donation to Infertile Spouses Act (IEDISA). Subsequently, the Iranian Legal Medicine Organization (LMO) and the Ministry of Health and Medical Education (MOHME) issued relevant guidelines for ARTs (9, 10, and 32). 

*Approach 4: Ethical Reasoning Using a Language Based on IBME Principles*

Some theorists of biomedical ethics, especially Sachedina, suggest a principle-rule based approach to ethical reasoning, which is similar to principlism as an important philosophical approach in MBME. This approach suggests that ethical reasoning in IBME should be done through some well-known Islamic principles generally used by Islamic jurists for juridical reasoning, because ‘Islamic jurisprudence’ (*Fiqh*) has been more established and disciplined than ‘Islamic ethics’ (discussed in approach 3). These principles include the principle of ‘public interest’ or ‘the common good’ (*Maslaha*), the principle of ‘no harm, no harassment’ (*La Darar wa la Derar*), the principle of ‘necessity’ (*Darura*), and the principle of ‘no hardship’ (*La Haraj*) (12 - 14). 

The principle of public interest (*Maslaha*) is an important principle that new rulings are generally derived from. *Maslaha* is also used to suspend earlier rulings out of consideration for public interest and welfare. However, some *Sunni* and *Shiite* jurists do not regard *Maslaha* as an independent source for ruling (12, 13). In *Shiite *or the* Mutazila *school of *Sunni, Maslaha *means providing benefits or preventing harms for people as much as possible. In the *Ash'ari* school of *Sunni, Maslaha *refers to what is assumed to be good according to *Sharia* laws, and protects the religion, lives (*Nufus*), reason (*Uqul*), lineage (*Nasl*), and property (*Mal*) of people (12, 13). 

Some Islamic principles are similar to the principles of MBME. For instance, the principle of ‘no harm, no harassment’ is similar to the two distinct principles of beneficence and non-maleficence. However, some significant Islamic rules are usually under-valued in MBME. According to Sachedina, one example is that the rule of consultation (*Shura*) is substituted by the principle of autonomy in MBME (13). 

Some of these principles have been applied in ethico-legal reasoning to justify abortion under specific circumstances. For instance, the principle of ‘protection against distress and constriction’ (‘Usr wa al-haraj’) is the main argument to justify abortion in case of a fetus younger than 4 months with severe malformation, retardation or abnormality that could lead to an unbearable suffering or difficulty for its mother. Similarly, the principle of ‘choosing the lesser harm’ between two harms is the main argument to prefer the mother’s life to that of the fetus, and can thus justify abortion when the mother is in a life-threatening condition (12, 36). 

**Challenges of general approaches to ethical reasoning in IBME**

In this section, some main challenges of each approach are discussed, but a comprehensive investigation of all the challenges is beyond the scope of this article.

*Challenges of Approach 1*

The first approach has the potential for effective communication between IBME and MBME discourses using the same language. One important challenge is how decision makers identify the lack of religious sensitivity or Islamic considerations about a specific issue, and consequently acknowledge that approach 1 is sufficient to deal with the problem; another challenge is to determine who should acknowledge this sufficiency.

*Challenges of Approach 2*

Similar to approach 1, approach 2 has the potential for communication between IBME and MBME discourses. Comparative analyses focusing on ethical reasoning using approach 2 can explore root causes leading to disagreements on the same issue in the two discourses. 

For a specific issue, approach 2 can be used appropriately based on the underlying assumptions which may be challenging. They are as follows:

     (i)   The issue is religiously sensitive. 

     (ii)  Some Islamic beliefs are so basic and common that in IBME, ethical reasoning about that issue can be based on those beliefs.

     (iii) Decision makers can take Islamic considerations about that issue into account using MBME ethics adjusted to the basic, common beliefs of Muslims.

     (iv) Decision makers do not need to seek *fatwas* to take Islamic considerations into account in ethical reasoning about that issue*.*

     (v)  Decision makers can recognize the above conditions for a specific issue.

Other important challenges are: who should be the decision makers; how they should acknowledge the sufficiency of approach 2 for ethical reasoning about that issue; how they should recognize the basic, common, beliefs of Muslims regarding ethical reasoning in that case; and how they should adjust MBME language to these basic, common beliefs of Muslims. 

*Challenges of Approach 3*

Approach 3 works well for issues that cannot be resolved using the two previous approaches. In this case, decision makers rely on *fatwas* to take Islamic considerations into account in ethical reasoning about a specific issue. In this age of communication and information, *fatwas* are easily found in the media worldwide. 

Although a general agreement exists among Islamic Jurists in many cases*, *sometimes different and even contradictory *fatwas* have appeared on the same issue during a given period of time, in various geographical locations, or even in the same country (37, 38). Diversity in *fatwas* results from different interpretations of *Sharia *and raises the problem of authority (i.e., which *fatwa* should be followed). This causes the most important challenge of approach 3: how healthcare professionals can resolve the conflicts among themselves or conflicts with their patients in the presence of diverse *fatwas*. 

*Fatwas* on abortion can be considered as an instance of diversity in Islamic *fatwas*. Abortion is generally prohibited in various schools of Islamic jurisprudence. After the 120^th^ day of gestation (when according to Islam the ensoulment occurs), Islamic jurists unanimously agree that therapeutic abortion is permissible only if a life-threatening condition places both mother and fetus in serious danger. However, abortion before the 120^th^ day of gestation is an area of disagreement. As a case in point, the majority of Shiite jurists do not permit abortion at any time unless an acceptable reason exists (e.g., a life-threatening condition or an unbearable suffering (*Usr wa Haraj*); however, a majority of *Sunni* Muslims, the *Hanafies*, permit an abortion up to the 120^th^ day of gestation, whereas most *Malikies* categorically prohibit abortion (37). Variety also exists in Islamic positions on abortion for non-medical reasons, for instance a woman’s physical or mental health, fetal impairment, social or economic reasons, and also in the case of incest or rape (36). 

Along with diversity in *fatwas* on abortion, there are different positions on the ‘abortion right’ in Islamic countries. A study drawing a cross-country comparison of abortion rights in Muslim-majority countries demonstrated substantial diversity: while 18 out of 47 countries are so conservative that they do not allow abortion under any circumstances except to save the mothers’ life, 10 countries allow abortion ‘on request’ before a specific period (37). 

Council (*Shura*) in *fatwa *may be a solution to address the challenge caused by diversity in fatwas. Some Islamic scholars have proposed to validate a *fatwa* by appointing groups of scholars qualified in Islamic jurisprudence. Issuing *fatwas* by such groups of scholars in institutional bodies has been a practical solution to the problem of diversity in *fatwas* since the1990s. Examples of these institutional bodies include: IOMS (The Islamic Organization for Medical Sciences, established 1994); IFA (The Islamic Fiqh Academy in Mecca, established 1977); IIFA (The International Islamic Fiqh Academy in Jedda, established 1981); and ECFR (The European Council for *Fatwa* and Research in Dublin, established 1997) (15). 

Additionally, we believe that in order to use approach 3 properly, theorists need to take a position on several challenging questions regarding the definition of *fatwa* in IBME discourse: 

     (i)   Whether juridical soundness of a biomedical practice makes it ethically sound.

     (ii)  Whether juridical soundness is a sufficient condition, necessary condition, both, or neither for ethical soundness in IBME. 

     (iii) Whether a fatwa must cover the full range of ethical concerns in biomedicine.

     (iv) Whether the juridical permissibility of a biomedical practice in *fatwas* specifically reflects the non-negative general view of Islam on that issue, i.e., ethical concerns need to be evaluated by Muslim biomedical ethicists.

*Challenges of Approach 4*

In our opinion, the workings of this approach are an excellent starting point to theorize and provide simple, effective models for ethical reasoning in IBME. However, it is necessary to address several important challenges to develop this approach as seen below:

     (i)   The different *Shiite* and *Sunni* juridical schools (*Hanafi, Hanbali, Maliki*, and *Shafi’i*) do not agree on the principles and how they are deduced from basic, rationalistically established ethical theories in Islam, according to Sachedina (12, 13). 

     (ii)   This approach has adopted Islamic ethico-legal reasoning as an integrated reasoning in Islamic discourse. Such reasoning works under two assumptions: 1) juridical obligations are necessarily consistent with ethical obligations, and 2) The aim of juridical obligations is to guarantee ethical obligations (12, 13). These assumptions are not accepted by all Islamic scholars.

     (iii)  It is unclear how these principles and rules have been selected from all Islamic juridical laws. In addition, the order of priority and the process of prioritization in the context of a moral dilemma are not determined. 

     (iv)  The agent who should use these principles and rules to make ethically sound decisions in IBME is not well-defined, and it is not clear if it is biomedical ethicists, healthcare professionals, or both. Whether these principles or rules are user-friendly enough to be applied by the moral agent is unknown as well. 

     (v)   Sachedina has made serious attempts to explore “distinctly Islamic, and yet cross-culturally communicable, principle-rule based deontological-teleological ethics that is operative in the Muslim legal-moral culture in assessing moral problems in IBME” (13). Nevertheless, it is yet unclear how this theory integrates principlism, deontology and teleological ethics, or how these principles can be “distinctly Islamic” and at the same time “cross-culturally communicable”(13), and if these two concepts are consistent. Likewise, these Islamic principles and non-religious ethical principles must be practically weighed and balanced to make decisions in biomedical practice, which is yet another issue that needs to be determined. 

## Conclusion

In this article, authors identified and conceptualized general approaches to ethical reasoning used in IBME discourse. These approaches include: 1) ethical reasoning using a language in common with MBME language; 2) ethical reasoning using MBME language adjusted to the basic, common beliefs of Muslims; 3) ethical reasoning using a language based on fatwas; and 4) ethical reasoning using a language based on Islamic biomedical ethics principles. 

This conceptualization is founded upon the distinctions drawn for a theoretical, heuristic purpose. It should be noted that in practice, developing arguments and decision-making concerning a specific subject should not be restricted to any one of these approaches, and a combination of these approaches is usually used for most issues. This is similar to other formal distinctions where the conceptually distinguished entities always exist in reality together. 

 To conceptualize general approaches to ethical reasoning in IBME, authors made an inductive distinction by review and content analysis of existing literature, as well as comparison between the languages of IBME and MBME. However, this review, content analysis and comparison have not been systematic, which is the biggest limitation of our work, and therefore this conceptualization may be regarded a tentative, starting point.

We believe that in order to develop a coherent theory for ethical reasoning in IBME, there is still a lot of work that needs to be done. To describe existing approaches to ethical reasoning in IBME and to prescribe new approaches, theorists must formulate more generic concepts and address the challenges of those approaches. This theorization requires further multidisciplinary work by biomedical ethicists, Islamic scholars, humanity and biomedical scientists, healthcare professionals, and laypersons from Muslim population.

## References

[1] Zalta EN The stanford encyclopedia of philosophy.

[2] Abbasi M, Shamsi Gooshki E (2017). Islamic bioethics and secular bioethics and interaction between them. Bioethics and Health Law Journal (BHL).

[3] Sugarman J, Sulmasy DP (2010). Methods in Medical Ethics.

[4] Ashcroft R, Lucassen A, Parker M, Verkerk M (2005). Case Analysis in Clinical Ethics.

[5] Beauchamp TL, Childress JF (2001). Principles of Biomedical Ethics.

[6] Fieser J, Kraft V Internet Encyclopedia of Philosophy.

[7] Al-Eraky MM, Chandratilake M (2012). How medical professionalism is conceptualised in Arabian context: a validation study. Med Teach.

[8] Mashayekhi J, Madani M, Saeedi Tehrani S (2015). Ethical considerations on advance directives: an overview of the ethical and legal aspects in the context of Islamic teachings. Journal of Medical Ethics and History of Medicine.

[9] Abbasi M, Shamsi Gooshki E, Allahbedashti N (2014). Abortion in Iranian legal system. Iran J Allergy Asthma Immunol.

[10] Abbasi M, Shamsi Gooshki E, Fakour H (2013). Legal protection for biomedical ethics in islamic republic of iran. Medical Law.

[11] Larijani B, Zahedi F (2006). Changing parameters for abortion in Iran. Indian Journal of Medical Ethics.

[12] Sachedina A (2009). Islamic Biomedical Ethics: Principles and Application.

[13] Sachedina A (2007). The Search for Islamic Bioethics Principles.

[14] Hanson HY, Sheikh, Aziz, Gatrad, Abdal Rashid (2008). Principles of Islamic Bioethics. Caring for Muslim Patients.

[15] Arda B, Rispler-Chaim V Islam and Bioethics.

[16] Ghaly M (2016). Islamic Perspectives on the Principles of Biomedical Ethics.

[17] Zahedi F, Sanjari M, Aala M (2013). The code of ethics for nurses. Iran J Public Health.

[18] Mobasher M, Aramesh K, Aldavoud S (2008). Proposing a national ethical framework for animal research in Iran. Iranian Journal of Public Health.

[19] Zahedi F, Larijani B (2008). National bioethical legislation and guidelines for biomedical research in the Islamic Republic of Iran. Bull World Health Organ.

[20] Tavaokkoli SN, Nejadsarvari N, Ebrahimi A (2015). Analysis of medical confidentiality from the Islamic ethics perspective. J Relig Health.

[21] Zahedi F, Larijani B (2009). Common principles and multiculturalism. J Med Ethics Hist Med.

[22] Mobasher M, Aramesh K, Zahedi F, Nakhaee N, Tahmasebi M, Larijani B (2014). End-of-life care ethical decision-making: Shiite scholars’ views. J Med Ethics Hist Med.

[23] Zahedi F, Larijani B, Bazzaz JT (2007). End of life ethical Issues and Islamic views. Iran J Allergy Asthma Immunol.

[24] Zahedi F, Larijani B (2007). Cancer ethics from the Islamic point of view. Iran J Allergy Asthma Immunol.

[25] Mobasher M, Salari P, Larijani B (2012). Key ethical Issues in pediatric research: Islamic perspective, Iranian experience. Iran J Pediatr.

[26] Chamsi-Pasha H, Albar MA (2013). Western and Islamic bioethics: how close is the gap?. Avicenna J Med.

[27] Daar AS, Khitamy A (2001). Bioethics for clinicians: 21. Islamic bioethics. CMAJ.

[28] Weber AS Bioethical reasoning in Islam.

[29] Aksoy S, Elmali A (2002). Core concepts of the four principles of bioethics as found in Islamic tradition. Med Law.

[30] Mustafa Y (2014). Islam and the four principles of medical ethics. J Med Ethics.

[31] Atighetchi D (2006). Islamic Bioethics: Problems and Perspectives.

[32] Shamsi Gooshki E, Allahbedashti N (2015). The process of justifying assisted reproductive technologies in Iran. Indian J Med Ethics.

[33] Nasir MA (2011). The majelis ulama's fatwā on abortion in contemporary Indonesia. Muslim World.

[34] Larijani B, Zahedi F (2007). Ethical and religious aspects of gamete and embryo donation and legislation in Iran. J Relig Health.

[35] Larijani B, Zahedi F (2007). Ethical and religious aspects of gamete and embryo donation and legislation in Iran. Journal of Religion and Health.

[36] Hedayat KM, Shooshtarizadeh P, Raza M (2006). Therapeutic abortion in Islam: contemporary views of Muslim Shiite scholars and effect of recent Iranian legislation. J Med Ethics.

[37] Shapiro GK (2014). Abortion law in Muslim-majority countries: an overview of the Islamic discourse with policy implications. Health Policy Plan.

[38] Hessini L (2007). Abortion and Islam: policies and practice in the Middle East and North Africa. Reprod Health Matters.

